# On the Employment of the Prussic Acid, as a Local Application, in Impetigo

**Published:** 1822-02

**Authors:** Anthony Todd Thomson

**Affiliations:** Member of the Royal College of Surgeons in London; Surgeon to the Chelsea and Brompton Dispensary, &c.


### MEDEOGRAPHY.

ART. VII.—*On the Employment of the Prussic Acid, as a local Application, in Impetigo.* By ANTHONY TODD THOMSON, F.L.S. Member of the Royal College of Surgeons in London; Surgeon to the Chelsea and Brompton Dispensary, &c.

HAVING been convinced, by numerous experiments upon quadrupeds, and, also, from closely watching its effects upon the human body when taken into the stomach, that Prussic acid is a direct sedative, and diminishes, more suddenly and effectually than any other sedative, the sensibility of nerves, I was induced to investigate its powers as a local application, in cutaneous affections accompanied with great itching and irritability. I am now induced to publish the result of my experience, in order that the attention of the profession may be directed to this employment of Prussic acid; and that its claims as an external remedy may be justly appreciated.

The first disease which I shall mention, as being more rapidly benefited by the external application of Prussic acid than by any other remedy, is IMPETIGO. In this affection, the discharge, which exudes from under the flaky crusts that characterize the eruption, is thin, and so extremely acrid, that it excoriates even the surrounding healthy skin, wherever it touches it, and thus spreads the disease to unaffected parts. The itching and tingling in the diseased limb is almost insupportable, particularly at night, when the patient is warm in bed; and little benefit is derived from the employment of any of the ointments or lotions commonly prescribed, or even from the internal exhibition of the largest doses of opium: indeed, the abraded surface, frequently, becomes so morbidly sensible, that the mildest applications cannot be borne. In this case, the torments of those afflicted with this disease are most lamentable; and, hitherto, the sympathy of the practitioner has been the only consolation he could offer to his patient.

Reflecting on the causes of this extreme irritability of the surface in Impetigo, I was led to conclude, that, if the nervous susceptibility of the diseased surface could be moderated, the discharge would necessarily become milder; and the excitement of the superficial vessels, which supply the discharge, being diminished, the exudation would not only be rendered milder, but be greatly diminished in quantity. No medicinal agent promised more certainly to effect such a favourable change than Prussic acid; and I had the satisfaction of witnessing my anticipations of its powers verified, in the first case which presented itself after my determination to employ it.

I was not, however, so sanguine as to imagine that any external remedy, even the most efficacious, could cure a disease depending on a disordered state of the general habit; but I confess that, after having seen the powerful sedative effects of the Prussic acid as a local application, I was in hopes that, by its internal exhibition at the same time that it was externally employed, the morbid irritability of the system, on which I conceived the disease to depend, would be brought under control by its means; and the healthy action of all the secerning and excretory organs readily restored. In this expectation, however, I was disappointed; and my next object was to investigate the state of the digestive organs, and to regulate the internal treatment by the result. I soon ascertained that, in several cases of the disease which came under my notice, that there was a superabundant acidity of the stomach; that the alvine evacuations were slimy, irregular, and denoted a deficiency of bile in the intestinal canal; and that a daily, although a slight, febrile paroxysm made its attack in the evening. With these facts clearly before me, I resolved to treat the disease in the following manner:—To alter the action of the liver, by throwing into the system a mercurial preparation, but in such doses as would enable it, also, in combination with an active purgative, to stimulate the orifices of the hepatic ducts, and thus increase the supply of bile to the duodenum; to neutralize, by an alkali exhibited in large doses, the acid present in the stomach; to improve the secretion of the stomach itself, by the alkali diminishing, as I conceive it always does, the irritability of the coats of that organ; and, at the same time, to moderate the local irritation, by the external employment of the Prussic acid. The two following cases are selected from many others, as a practical illustration of the success of this method of treating Impetigo, merely because they occurred in persons placed in opposite ranks of life; and, therefore, display the effects of the treatment under very different circumstances.

CASE I.

14*th February*, 1821.—James Exton, æt. 22, by trade a plasterer, and living in Clifford-row, Pimlico, was this day admitted a patient of the Chelsea and Brompton Dispensary. The patient was a stout man, of a sanguine temperament. The whole of the left leg, from the instep of the foot to the knee, was more or less covered with an inflammatory eruption, which, on a close examination, consisted of minute pustules surrounded by a red, glazed base; and, where they were unbroken, the pustules were filled with a yellowish fluid. In many places along the tibia, where the bone is covered by little more than the skin, this surface was excoriated, and the intermediate cuticle red, shining, and perforated with minute pores, from which a thin ichorous discharge poured out; while other parts of the surface were covered with thin, yellowish, flaky scales, turning up at the edges, and oozing out, from beneath them, the same ichorous discharge that was supplied by the broken pustules. The patient complained of the most intolerable itching and tingling of the limb, which completely destroyed his rest at night, and obliged him to rub and scratch the diseased part; although he was well aware that, by doing so, he only increased the evil. The pulse was small, but natural in point of quickness; the bowels were rather open than otherwise, and much disturbed with flatulence; but his appetite was good, and, to use his own language, "he would be quite well, were his leg but sound, and he could sleep at night." He could assign no cause for the disease, (which had, now, existed for more than six weeks,) unless it might have arisen from drinking cold porter, while he was in a state of perspiration from his work. He had tried a great variety of ointments, which had only increased the tingling and itching; and had taken a considerable quantity of purgative medicine.

As this was decidedly a case of *Impetigo sparsa*, I resolved to try the effect of the treatment which had suggested itself to my mind; and, accordingly, the following medicines were prescribed for him:

℞. Pilulæ Hydrargyri,
——- Hydrargyri Submuriatis compositæ, āā ʒss.
Extracti Colocynthidis, ʒj ss. Simul contunde, et divide massam in pilulas æquales xxx. quarum sumat j mane et ij nocte, quotidie.

℞. Extracti Sarsaparillæ, ʒiv.

Sodæ Subcarbonatis, ʒviij.

Infusi Cinchonæ, f. ℥xij. Misce ut fiat mistura, cujus cyathus sumatur ter quotidie.

℞. Acidi Hydrocyanici *(Prussici)*, f. ʒiv.
Spir. Vini rectificati, f. ℥j.
Aquæ Distillatæ, f. ℥x ss. Misce ut fiat Lotio, diligenter utenda.

He was ordered to be very regular in taking his medicines; to keep the whole of the leg constantly moist, by means of soft rags soaked in the lotion; and to live on a light animal diet, avoiding sweet things, raw and crude vegetable matters, spirits, and malt liquor.

17*th*.—The leg was much improved in appearance; the inflammation considerably abated, and the discharge lessened. The greatest relief had been experienced from the application of the lotion, in allaying the itching; and he had enjoyed two nights of good repose, which had much refreshed him. He was ordered to continue the same plan; which was pursued, with scarcely any variation, until the 19th of May, when he was discharged cured; the skin of the leg having recovered its natural colour, and every symptom of the disease having disappeared a week prior to that period.

On the 30th of May, however, James Exton was again admitted a patient of the Dispensary; the disease, of which he had been so recently cured, having attacked the other leg. On questioning him, I found he had been in the country, living rather freely amongst his relations. The eruption was extended over the front only of the leg, and much less formidable in its aspect than it had been in the other limb. His bowels were much confined; and his digestive organs were evidently more deranged than on the former occasion. The disease had appeared five days ago, and, as it was increasing, he had returned to Chelsea, for the purpose of again placing himself under my care.

℞. Pilulæ Hydrargyri Submur. comp. ℈ss.
Extracti Hyosciami, gr. xviij. Ft. pilulæ sex. Sumatur j omni nocte.

℞. Extracti Sarsaparillæ, ʒiij.

Sodæ Subcarbonatis, ℈vj.

Infusi Cinchonæ, f. ℥xij. Misce, sumantur cochlearia amp. iij. ter quotidie.

A brisk purgative was ordered to be first taken; and he was directed to bathe the limb, frequently, with tepid bran gruel, and to apply over it the Oxyd-of-Zinc ointment, spread on lint; as I was desirous of ascertaining how far the constitutional treatment would succeed without the aid of the local sedative.

*June 2d.*—The eruption is no better in appearance, and much more moist and irritable. Continue.

*6th.*—Nearly in the same state. Continue.

*9th.*—He now complained much of the increased itching and irritation, and begged hard to have the same lotion that had formerly been ordered for him. It was, accordingly, ordered; but with three fluid-drachms only of the acid; and the same internal treatment was continued.

*13th.*—He has found much relief from the use of the lotion, and the improvement is very apparent in the aspect of the eruption. He had been, however, so much purged, and his tongue was so much chapped and so sore, that I ordered him to discontinue the pills and the mixture last prescribed, and gave him the following:

℞. Pulveris Rhei, gr. viij.

——— Ipecacuanhæ comp. gr. vj. Pulvis quam primum sumendus.

℞. Misturæ Cretæ, f. ℥vss.

Pulveris Ipecacuanhæ comp. ℈j.

Tincturæ Catechu, f. ʒiv. Misce ut fiat. Mistura, cujus Cochlearia amp. ij 6tis horis sumantur.

20*th*.—His bowels being now quite comfortable, he returned to the use of the pills and mixture ordered on the 30th of May; which he continued, with the lotion, until the 20th of July, when, every appearance of disease having left him for a fortnight, he was discharged; and has since continued perfectly well.

The same plan of treatment has been pursued in every case of Impetigo that has since come under my care at the Dispensary; and, although these have been numerous, and some of them cases of very long standing, yet I have not met with one instance which has not, sooner or later, yielded to its power.

CASE II.

J. C—ll, esq. applied to me, in the beginning of last year, on account of an eruption which had appeared on his shin, and which he attributed to the skin having been fretted by the stirrup-leather in riding. On examination, I found an eruption on the shin, in the form of two distinct spots, one about three inches in length and two in breadth, and the other an inch in diameter in both directions. Each spot consisted of a highly-inflamed, rough, scaly zone of an irregular form, surrounding a glazed, red portion of skin, perforated by numerous, very minute, ulcerated pores, oozing a thin, acrid ichor; which, in some places, had concreted into thin flaky scales, turned up at the edges, and of a greenish-yellow colour. Beyond the circumscribing zone, several small clusters of pustules were perceptible, which, extending in every direction, appeared disposed to coalesce: and in fact, by the union of such clusters, new zones were successively formed on the outside of the old, and the disease gradually spread itself over the greater part of the lower limb. The pulse was rather small and quick, the bowels were irregular, and the tongue was red and chapped; but Mr. C. did not, otherwise, complain of any constitutional disturbance.

From the nature of the eruption and its progress, (for I was informed it had commenced in two small clusters of pustules,) there was no doubt that the disease was the *Impetigo figurata* of Willan. Mr. C—ll had paid little regard to it, until the spots began to enlarge, and had done nothing more than washing the affected parts with Goulard-water, previous to the period at which I was consulted, which was about six weeks after the first appearance of the eruption. It is unnecessary to detail the variety of treatment, both local and general, which was resorted to for several months. After every change of remedies, the symptoms apparently abated, and the skin seemed about to acquire its healthy state; but these favourable appearances were only transitory and fallacious, and, after any plan, which at its commencement promised a satisfactory result, had been pursued for a short time, a relapse always took place; and the symptoms, instead of appearing improved, seemed to have acquired only more virulence by their suspension. Finding all the usual means unsuccessful, I advised Mr. C—ll to go to Worthing, to try the tepid sea-water baths. He remained there two months, and was certainly much benefited by the baths; but, soon after his return to London, the disease recurred, and I was again consulted as to the means necessary for its removal.

Having now had sufficient proofs of the efficacy of Prussic acid in impetiginous affections, I told him there was every reason for thinking he would soon get well. He agreed to try any plan I might suggest; but, as he had previously taken much medicine, he trusted the remedies would consist chiefly of external applications. I promised to limit the internal treatment as much as possible; and therefore, on the 14th of September, prescribed for him the following pills and lotion:

℞. Pilulæ Submuriatis Hydrargyri, ʒj.
——— Aloes cum Myrrha, ʒjss. Simul contunde et divide in pilulas æquales xxx. Sumantur iij omni nocte.

℞. Acidi Hydrocyanici, f. ʒij.

Aquæ Distillatæ, f. ℥v. ss.

Spir. Vini rectificati, f. ʒiij. Misce ut fiat lotio parti ægrotæ applicanda.

In a very few days, the itching and tingling, which had again become very troublesome, abated; and at this time (18th January,) the disease is completely removed.

Were I anxious to multiply cases to illustrate the powerful sedative effects of the Prussic acid in Impetigo, the case-books of the Chelsea and Brompton Dispensary furnish ample means. In one of the cases detailed there, the disease had existed twelve years; and the female, a servant, had been two years attending the Dispensary, but in the last four months only had she received any permanent benefit, and that arose from the use of the Prussic acid, which at length effected a perfect cure. I am, however, less desirous to detail my own experience, than to direct the attention of the profession to so powerful an agent in the treatment of a class of diseases which has hitherto baffled almost every means which have been tried for their removal.

I am well aware that the price of Prussic acid is a great objection to its general employment in Dispensary practice; and I regret that my experience does not permit me to say that its place can be supplied by any of the vegetable substances which, from their flavour, evidently contain Prussic acid. At least, I have been disappointed in my trials of decoctions of Laurel-berries, the Bitter-almond emulsion, and the strongest infusions of Peach, Nectarine, and Laurel leaves. I propose trying the Cyanate of mercury, which is more easily prepared, and is, consequently, less expensive than the Prussic acid.

In another communication, I shall detail my experience of the utility of Prussic acid in *Acne rosacea*, and some other cutaneous eruptions.

